# 3D printing: Balancing innovation for sustainability with emerging environmental and health risks

**DOI:** 10.1016/j.isci.2025.113185

**Published:** 2025-07-23

**Authors:** Andi Alijagic, Damir Suljevic, Magnus Engwall, Eva Särndahl

**Affiliations:** 1 Inflammatory Response and Infection Susceptibility Centre (iRiSC), Örebro University, SE-701 82 Örebro, Sweden; 2 Faculty of Medicine and Health, School of Medical Sciences, Örebro University, SE-701 82 Örebro, Sweden; 3 Man-Technology-Environment Research Center (MTM), Örebro University, SE-701 82 Örebro, Sweden; 4 Department of Biology, Faculty of Science, University of Sarajevo, Zmaja od Bosne 33-35, 71 000 Sarajevo, Bosnia and Herzegovina

**Keywords:** Public health, Environmental health, Industrial engineering

## Abstract

The rapid rise of 3D printing, both in industrial and home settings, presents emerging health and environmental risks. While 3D printing enhances sustainability by reducing waste and optimizing resource use, its impact on human health remains poorly understood. The use of metals and polymers linked to health risks, coupled with the release of inhalable particles and volatile organic compounds, raises concerns about respiratory and systemic effects. The absence of clear guidelines creates high public demand for information and limits safe implementation, particularly in schools and homes where millions of 3D printers are expected by 2030. Additionally, improper disposal of 3D printing polymer materials may exacerbate plastic pollution. This article proposes the perspective of a structured risk assessment framework set on particle emissions from industrial 3D printing. It will offer a practical tool to bridge current knowledge gaps and to inform safe practice and policy development, because immediate action is necessary to balance innovation with safety.

## Graphical abstract

**Figure:**
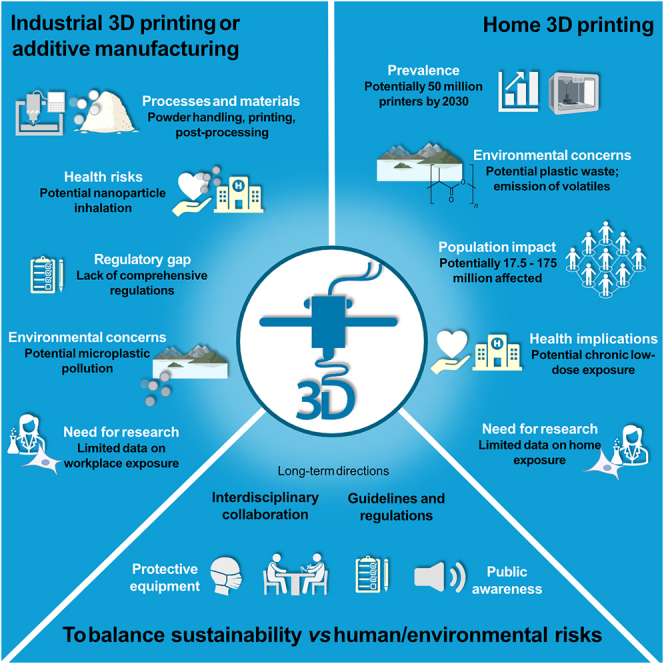

## Introduction

**Figure 1:**
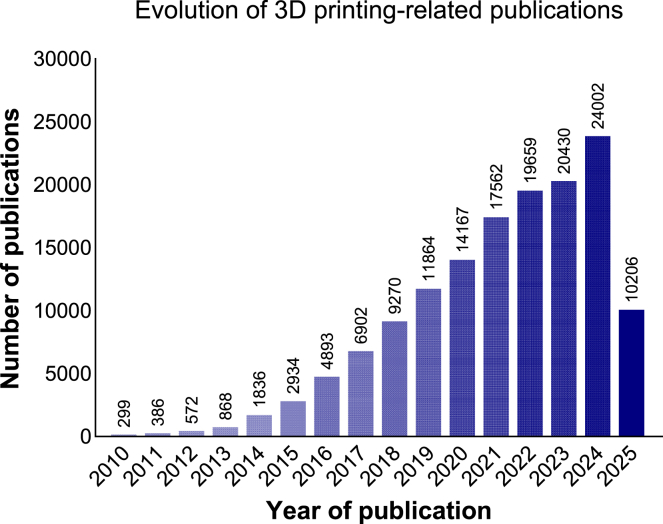
Evolution of 3D printing-related publications over the last 15 years The bar chart shows the annual number of publications from 2010 to 2025 retrieved from the Web of Science Core Collection using a combined search for terms related to 3D printing: “*3D print*,” “*3D printing*,” “*3D printing technology*,” “*3D-printing*,” “*additive manufacturing*,” and “*three-dimensional printing*.” A total of 145,850 publications were identified, highlighting a sharp increase in research output.

3D printing was originally developed by Charles Hull in the early 1980s under the name Rapid Prototyping or Solid Freeform Technology and has been in use for over three decades.^1^ Figure 1 illustrates the publication trend related to 3D printing over the past 15 years, highlighting a rapid surge in technological development over the last decade. Notably, this trend saw an additional increase during the COVID-19 pandemic, driven by the urgent need for decentralized and on-demand production solutions.^2^

The rise of industrial and home 3D printing technologies presents both a potential breakthrough in innovation for sustainability, while at the same time emerge as a risk for sustainability in regard to health and environment. 3D printing’s promise lies in its ability to revolutionize the production of objects with complex geometries as well as to reduce waste, optimize resource use, and lower energy consumption compared to traditional manufacturing,^3^^,^^4^^,^^5^ whilst also offering the opportunity for advancing material recycling and circularity.^6^^,^^7^^,^^8^^,^^9^ The European Union (EU) has recognized industrial 3D printing, also known as additive manufacturing (AM), as a Key Enabling Technology (KET) (STOA, 2021),^10^ underscoring its role in transforming industries and generating new markets (see Box 1 for their brief explanation).

Box 1 **Key enabling technologies**

Key enabling technologies (KETs) —a group of six technologies defined by the EU’s High-Level Group on industrial technologies: micro and nanoelectronics, nanotechnology, industrial biotechnology, advanced materials, photonics, and advanced manufacturing technologies— increase industrial innovation to address societal challenges and creating advanced and sustainable economies (STOA, 2021). KETs have the potential for application in virtually all sectors and industries, including aeronautics, automotive, engineering, chemicals, textiles, space, construction, healthcare, and agriculture.

3D printing, particularly in industrial settings, employs a range of complex processes and materials that can release micro and nanoscale particles during various operations. For some of the materials, the compositions have well-characterized health risks,^11^^,^^12^ whereas for others, their potential negative impacts on health and the environment are today unknown or rely on knowledge adopted from settings with, e.g., lower exposures and with other chemical combinations and particle sizes.^13^^,^^14^ The particles and chemicals can enter the body by inhalation or ingestion, which potentially may lead to disease by affecting the respiratory system but also by entering the circulation and subsequently accumulating in organs.^15^^,^^16^ In addition to industrial setting, 3D printing technology is entering households and schools—making the concerns on health afflicting a substantial part of society.

While 3D printing is widely seen as a driver of sustainable manufacturing due to its resource efficiency and digital flexibility, its environmental and health risks, particularly from particle and chemical emissions, represent a largely overlooked sustainability challenge. The 3D printing community has raised concern as they fear potential occupational health risks that could result in a future tragedy, as was the case in the past with silica/quartz, coal, and asbestos,^17^ but data on health effects caused by 3D printing-related particle emissions are currently limited, thus preventing conclusions to be made.

Guided by the growing body of evidence on health effects caused by ambient exposure and from particle exposure in traditional industries, studies —*in vitro*, human *in vivo*, epidemiological, and in the area of occupational health— all tell the story of particle toxicity as an emerging occupational health risk^18^^,^^19^^,^^20^^,^^21^ and to cause disease.^22^^,^^23^ In addition, 3D printing generates nanoparticles that may pose distinct and potentially harmful health effects of their own.^24^ Another important consideration is that much of the existing research is conducted within narrow scopes —focusing on individual aspects such as exposure levels, particle characterization, or toxicity— without connecting these elements to broader health outcomes. While of importance, these isolated pieces of data need to be put into a larger perspective to understand potential hazards for human health.

Combined with the current lack of knowledge on safe handling practices for emitted particles, the 3D printing sector remains without adequate health and safety assessment tools for emissions across the process chain, and national guidelines or occupational exposure limits (OELs) for nanoparticles are still lacking in most countries. In addition, lack of information regarding improper disposal of 3D printing materials will impact the environment and further impose the environmental pollution crisis.^5^^,^^25^ For the rapid advancement and widespread adoption of 3D printing technologies, particularly in home settings, societal demands to scrutinize its broader impact on human and environmental health are therefore high up on the agenda.^26^ This calls for interdisciplinary collaboration between academy, industry, and society at large to address these challenges with the aim of finding sustainable solutions.

This perspective aims to develop and propose a generalizable risk assessment framework focusing on unintentional particle emissions in industrial 3D printing and to discuss its relevance for human health protection and sustainable technological advancement. By directly addressing a critical knowledge gap in sustainable innovation, this framework supports responsible development and ensures that the expansion of 3D printing technologies aligns with both public health protection and environmental sustainability.

## The hidden risks of 3D printing

3D printing is a technology that in the last decade has revolutionized the industry sector as well as other parts of society, including schools, and continues to do so by its ability to create solid objects of virtually any geometry. The technique uses materials including various metallic, plastic, ceramic, and composite powders as well as plastic filaments,^27^^,^^28^^,^^29^^,^^30^^,^^31^ which are feedstocks of distinct size, shape, and chemistry. Micro and nanoscale particles can be released from these materials during operations, such as powder handling, printing, post-processing, machine maintenance, and cleaning,^32^^,^^33^^,^^34^^,^^35^ and will, to different extents, be emitted into the 3D printing work environment, offices, schools, and households with 3D printers. In industrial settings, the existing air measurements reveal that different 3D printing processes entail the emission of large numbers of (nano)particles, reaching up to 500,000 particles/cm^3^ inside the printer hood, meaning that 3D printing workers risk to be repeatedly exposed daily to (nano)particles with largely unknown biological effects.^36^ Notably, Gu et al.^37^ reported emission rates as high as 1.7 × 10^11^ particles per minute for certain materials and printers, further underscoring the potential for high exposure scenarios. Even if some of the materials used in 3D printing have compositions with well-characterized health risks, such as silica, nickel, cobalt, or chromium,^38^ most information on particulate matter is currently originating from studies on ambient exposure^39^^,^^40^ or from particle exposures at traditional industries, like foundries, hard metal industries, and construction.^41^^,^^42^^,^^43^^,^^44^^,^^45^ However, occupational exposures involve higher levels of particulate matter compared to ambient air pollution and with varying chemical compositions, and of significance, 3D printing generates high amounts of nanosized particles; conditions which will potentially result in more severe health effects.^46^

If nanoparticles generated during 3D printing follow the same trajectories and will give rise to similar effects as observed from ambient pollutants, these particles, due to their small size and unique physicochemical properties, may pose significant health risks if inhaled or ingested.^15^^,^^16^ They may deposit in the respiratory tract, enter the circulatory system and accumulate in organs throughout the body, potentially leading to respiratory, cardiovascular, and neurodegenerative diseases,^18^^,^^47^^,^^48^ cancer, and reproductive system toxicity.^49^^,^^50^

3D printing technology is rapidly entering tens of millions of households and public spaces worldwide. While reliable year-by-year projections and precise estimates of the number of individuals potentially affected by emissions remain difficult due to limited and inconsistent data, it is nonetheless crucial to consider the health implications for users and their households – especially given that an estimated 50 million home 3D printers are projected to be sold annually by 2030 (3D printing industry, 2024).^51^ Given the potential for widespread exposure, even if only a fraction of these devices releases hazardous particles, the scale of the impact could be substantial. For instance, if 10% of these printers produce emissions that affect users, this could involve 5 million households globally. Assuming an average household size of 3.5 persons, up to 17.5 million individuals could be exposed to potentially harmful emissions from home 3D printing. In a worst-case scenario, this number could rise to 175 million individuals. Such a scenario could indeed pose a significant threat to human health. However, these numbers should be interpreted in light of varying exposure conditions. Factors such as printer type, use frequency, enclosure, and ventilation significantly affect actual exposure levels (NIOSH, 2023).^52^ Nevertheless, the potential impact is substantial for several reasons, including a considerable public health burden, strain on healthcare systems and resources, and potential increases in healthcare costs.

Data on health effects caused by 3D printing-related particle emissions are today limited. With 3D printing being a new technology, the effects of long-term exposure of low doses of nano-sized particles are not yet possible to study but are an additional exposure scenario of highest importance by being the most likely in 3D printing settings. Whereas existing studies primarily focus on occupational exposure in industrial settings,^21^^,^^36^^,^^53^ similar risks may be present in home and public space environments.^54^ The likelihood for the chronic, low-dose exposure to nanoparticles in residential settings is a significant concern, as these particles can have cumulative and long-term health effects.^55^^,^^56^^,^^57^ This is of particular concern for vulnerable groups, including children,^58^^,^^59^ pregnant women, and individuals with pre-existing respiratory (e.g., asthma)^60^ or cardiovascular conditions. In addition to the release of inhalable particles, also volatile organic compounds (VOCs) are formed in 3D printing.^37^^,^^61^^,^^62^^,^^63^ As the 3D printing technology is becoming increasingly common in educational settings, teachers and other staff at schools and after-school programs are reaching out to academia with questions and growing concerns about the lack of information on safe 3D printing practices.

The environmental impact of 3D printing materials, especially feedstock powders composed of microscale particles used in industrial 3D printing, should not be overlooked. The production and disposal of feedstock powders and prints may contribute to environmental pollution if not managed properly.^25^^,^^64^ For instance, improper disposal of polymer-based powders, containing primary microplastics, can lead to unintended microplastic pollution, impacting both terrestrial and marine ecosystems by polluting oceans and landfills, harming wildlife, increasing emissions of greenhouse gases, and further exacerbating the plastic pollution crisis.^65^^,^^66^^,^^67^

**Figure 2:**
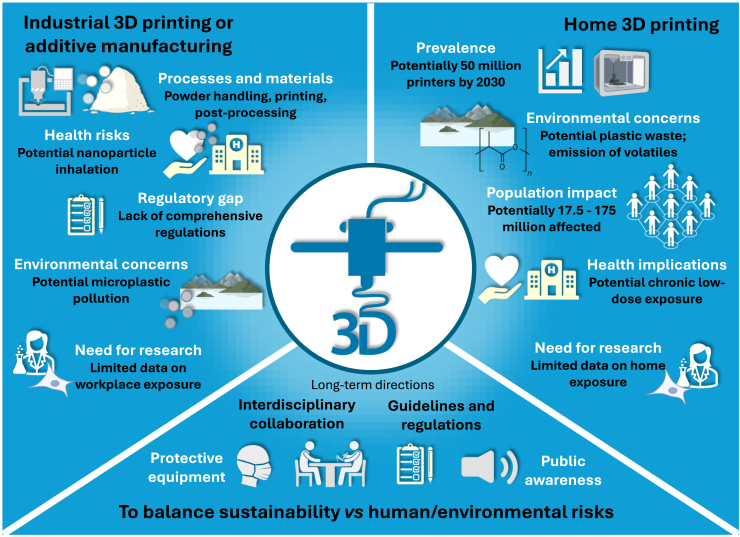
Industrial and home 3D printing in the context of emerging environmental and health risks This figure illustrates the growing use of 3D printing in both industrial and home environments, highlighting potential sources of particle and chemical emissions. It also emphasizes the associated environmental and human health risks, particularly in settings with insufficient safety measures or limited awareness of exposure hazards.

Addressing the concerns raised by the 3D printing technologies requires interdisciplinary collaboration to develop comprehensive global guidelines and regulations for 3D printing safety, both in industrial and home settings. Research should focus on characterizing the particle emissions from various 3D printing processes and materials, assessing their health risks, and establishing safe exposure limits and information on *which*, *what*, and *where* in regard to these exposures. Additionally, public awareness campaigns and education on safe practices for home 3D printing are essential to prevent adverse health effects. The challenge to balance the development of new technologies without running the risk of jeopardizing human and environmental health demands bilateral communication and knowledge transfer within society, involving all actors - including industry, academia, healthcare, and politicians (Figure 2).

## The rise of 3D printing: Pushing the sustainability frontier

3D printing has emerged as a transformative technology over the past decade,^68^^,^^69^^,^^70^ reshaping industries and offering unprecedented opportunities for sustainable innovation. Over the past decade, research on 3D printing has surged, with 145,850 original articles (June 10, 2025; Figure 1) indexed in the Web of Science Core Collection. Unlike traditional subtractive manufacturing methods, which involve carving material away from a larger block, 3D printing builds components layer by layer, utilizing only the material needed.^71^^,^^72^^,^^73^ This process minimizes waste, setting a new benchmark for sustainable production.^74^ By leveraging 3D models sliced into thousands of layers, 3D printing enables the precise deposition of material, reducing the environmental footprint of manufacturing processes across various sectors.^3^^,^^4^^,^^75^^,^^76^ However, the sustainability of 3D printing must also account for the energy-intensive processes required to produce feedstock materials, in particular metal powders, including melting feedstock, atomizing, collecting, classifying, storing, and distributing the material for use. While 3D printing minimizes material waste, these steps add to its environmental footprint and should be factored into its green potential.^77^

3D printing technologies encompass a wide range of processes (see Box 2 for their brief explanation) and materials, each offering unique capabilities.^78^^,^^79^^,^^80^ Among the most widely used is the laser powder bed fusion (L-PBF) process, which melts and fuses metal or polymer powders with remarkable precision.^81^^,^^82^^,^^83^^,^^84^ These advancements allow for the creation of complex geometries and lightweight structures previously unattainable through conventional methods.^85^ Current capabilities include printing with polymers, aluminum alloys, titanium, ceramics, and composites, achieving layer thicknesses as fine as 20–100 μm.^79^^,^^80^^,^^83^^,^^86^

Box 2 **Glossary of 3D printing techniques**

Powder bed fusion (PBF – metal or polymer) – A process where thermal energy from a laser or electron beam is used to selectively fuse regions of a powder bed. Laser powder bed fusion (L-PBF): Uses a laser to melt metal or polymer powders layer by layer to produce highly detailed parts. Electron beam melting (EBM): A metal-focused variant using an electron beam in a vacuum, typically for dense, high-strength components. Selective laser sintering (SLS): Common for polymers; a laser sinters powder without fully melting it, enabling support-free geometries.

Material extrusion – A technique where thermoplastic material is heated and extruded through a nozzle to build objects layer by layer.

Vat photopolymerization – A resin-based process where a liquid photopolymer is selectively cured using a light source. Stereolithography (SLA): Uses a UV laser. Digital Light Processing (DLP): Uses a digital projector to cure each layer more quickly.

Binder jetting – A method where a liquid binder is deposited selectively onto a powder bed to bond particles layer by layer. Post-processing often includes sintering or infiltration.

Material jetting – Similar to inkjet printing, this technique deposits droplets of build material (typically photopolymers or waxes), which are then cured with UV light. Allows high-resolution, multi-material printing.

Cold spray additive manufacturing (CSAM) – A solid-state process where metal powders are accelerated at high speed by a gas jet and deposited onto a substrate to form near-net-shape parts.

3D printing is uniquely positioned to address some of the sustainability grand challenges faced by modern manufacturing. By reducing material waste and enabling resource-efficient production, 3D printing contributes to a significant reduction in energy consumption and greenhouse gas emissions across product life cycles. For example, model calculations predict that industrial 3D printing could lower total primary energy supply by 2.54–9.30 exajoules (EJ) and reduce CO_2_ emissions by 130.5–525.5 million metric tons by 2025.^3^ This potential extends to high-value, low-volume production sectors, such as aerospace and medical device manufacturing, where 3D printing’s ability to produce complex and customized parts significantly lowers resource demands and CO_2_ emissions.

3D printing’s ability to decentralize production shifts supply chains toward localized and digital models, reducing the environmental costs of transportation and supporting circular economies.^87^ Unlike injection molding, which requires expensive molds and tooling, 3D printing involves relatively low fixed costs, making it economically viable for small production runs and niche markets. Moreover, 3D printing’s capacity to recycle 95%–98% of waste material in metal applications^83^ further supports its alignment with sustainable manufacturing principles.

By combining reduced material waste, energy efficiency, and digital innovation, 3D printing exemplifies the potential for manufacturing technologies to align with sustainability goals.^87^ However, realizing this potential requires addressing challenges such as resource availability, hazardous emissions, and waste management within the life cycle of 3D printing processes. As industries continue to adopt 3D printing on a large scale and as 3D printers enter homes and public spaces, ensuring its environmental and social benefits will be essential to fulfilling its promise as a cornerstone of sustainable manufacturing.

## Industrial 3D printing: Emissions and associated health impacts

**Figure 3:**
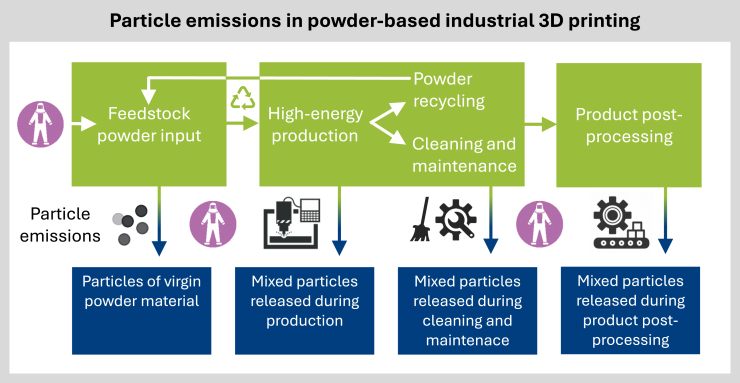
Powder-based industrial 3D printing and particle “leaking” spots throughput the powder life cycle Powder input largely results in emission of micron-sized feedstock particles, while high-energy production, powder recycling, machine cleaning and maintenance, and product post-procession result in the emission of feedstock and unintentionally generated (nano)particles.

The use of fine metal or polymer powders and high temperature processes in industrial 3D printing generates particulate matter (PM) throughout the powder life cycle (Figure 3) that poses risks to human health and the environment.^34^^,^^53^ In powder-based industrial 3D printing, feedstock particles typically range from 25 to 150 μm in size and require specialized storage due to their physical and chemical properties.^88^ During the printing process, micro and nanoscale particles are unintentionally released, contributing to potential airborne exposure.^34^^,^^89^^,^^90^ The various metals and alloys commonly used in 3D printing may pose additional health risks. For instance, titanium, valued for its biocompatibility and inertness, mechanical strength, and corrosion resistance, is commonly used in 3D printing but releases particles during processing. These particles cannot be metabolized by the human body and will instead accumulate, and may reach toxic levels.^91^ The respiratory tract is a primary target for PM exposure. Deposition of particles in alveoli exceeds 50% for inhaled nanosized particles around 20 nm in size but drops below 20% for larger particles around 200 nm.^92^ Nanoparticles exhibit lower lung clearance efficiency compared to larger particles, as observed in rodents.^93^ For example, 29 nm TiO_2_ nanoparticles induce pulmonary inflammation in rats, whereas submicron-sized particles (250 nm) do not.^94^

From a health and safety perspective, 3D printing techniques that employ metal or polymer (primary microplastic) powders as a feedstock are particularly concerning.^95^ For example, in the cold spraying process, metal powder particles are plastically deformed at high speeds onto substrate surfaces, where they bond together.^96^ Particle deformation may also alter their toxicity profiles.^34^ Despite advances in these techniques, some powders may escape containment chambers, calling for further health and safety evaluations.^53^ Notably, metal processing techniques, including silver refining, have been found to release significant quantities of metals and nanoparticles, with emissions of antimony, selenium, and zinc increasing by up to 1,000-fold compared to background levels.^97^ Similarly, nanoparticles generated during 3D printing remain airborne for extended periods due to their small size, low density, and slow settling rates, increasing the likelihood of inhalation exposure, particularly in industrial settings where ventilation may not effectively remove them. In unventilated spaces, these particles can remain suspended for several hours, with deposition rates decreasing as humidity rises.^98^ Additionally, nanoparticles can be resuspended from surfaces by air currents, mechanical vibrations, or human activity, further prolonging exposure risks in occupational environments.

**Figure 4:**
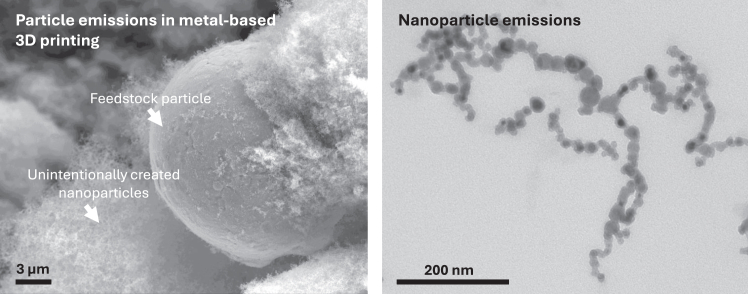
Examples of unintentional particle emissions in metal 3D printing Left panel: scanning electron microscopy (SEM); right panel: transmission electron microscopy (TEM) images of (nano)particles unintentionally generated during industrial 3D printing with iron-based powders using laser powder bed fusion. Images courtesy of Dr. Patrik Karlsson, Örebro University, and Prof. Oldřich Benada, Institute of Microbiology, Czech Academy of Sciences.

Research on the health and safety aspects of particles emitted from 3D printing are limited (680 original articles (April 07, 2025) indexed in the Web of Science Core Collection) and has yielded conflicting results. This poses two key risks: (1) the risk that the industry may not reach its full potential, and (2) the risk of adverse impacts on both environmental and human health. For example, Karlsson et al.^14^ observed limited cytotoxic, genotoxic, and oxidative stress effects in cultured human lung cells exposed to particles released from nickel-based alloys during 3D printing. In contrast, Alijagic et al.^99^ demonstrated significant cytoskeletal and mitochondrial disruptions, oxidative stress, and membrane remodeling in lung epithelial cell-macrophage co-cultures exposed to 3D printing-emitted iron-based nanoparticles (Figure 4). Vallabani et al.^100^ reported low acute genotoxicity of condensate/spray particles generated during selective laser melting (SLM) using nickel, chromium, steel, and titanium-based alloys at doses of 10–100 μg/mL. Similarly, metal particles released in artificial lysosomal fluid (ALF) from stainless steel and tool steels showed no significant cytotoxic or genotoxic effects in human bronchial epithelial cells, although certain powders induced pro-inflammatory cytokine secretion.^101^ Assenhöj et al.^13^ reported low worker exposure to inhaled dust and metals during 3D printing tasks but noted potential impacts on kidney function and respiratory tract inflammation. Polyamide-12 microplastics, used as a particulate feedstock in polymer 3D printing, also pose potential health risks, as they were found to increase levels of the pro-inflammatory chemokine interleukin-8 (IL-8/CXCL-8) in human primary macrophages over a two-week exposure.^102^ In addition to particulate matter, powder-based polymer feedstocks contain chemical additives and unreacted monomers that can leach or be emitted, and their health impacts remain largely unknown. A recent study showed that polyamide-associated chemicals modulate the kynurenine pathway and exhibit antiandrogenic activity.^102^ In conclusion, these studies provide important but still fragmented data by investigating different kinds of cells, particles, conditions, etc. making it hard to draw conclusions. In addition, the data are mostly reflecting acute exposure effect, whereas data from long-term exposure is today missing. For definitions of key toxicological terms such as cytotoxicity, genotoxicity, and oxidative stress, see Box 3.

Box 3 **Glossary of key toxicological and biological terms**

Antiandrogenic activity – A substance’s ability to block or reduce the effects of male hormones (androgens).

Carcinogen – A substance that can cause cancer.

Carcinogenesis – The process by which normal cells turn into cancer cells.

Clearance efficiency – How well the body can remove particles or substances, such as from the lungs.

Cytoskeletal and mitochondrial disruptions – Damage or changes to the cell’s structural framework (cytoskeleton) and energy-producing parts (mitochondria).

Cytotoxicity – The property of particles or chemicals to cause damage to or kill living cells.

Endocrine disruption – Interference with the normal function of the hormonal (endocrine) system, which can lead to developmental, reproductive, neurological, and immune problems in both humans and wildlife.

Eosinophilic inflammation – A type of inflammation involving eosinophils, a type of white blood cell often active in allergies or asthma.

Epithelial cell – A type of cell that lines the surfaces of the body, such as skin, airways, and intestines.

Genotoxicity – The ability of particles or chemicals to damage the genetic material (DNA) within a cell, potentially leading to mutations, cancer, or other health effects.

Immune sensitizer – A substance that can trigger the immune system to react, potentially causing allergies.

Immunosuppressed – A state where the immune system is weakened or not working properly.

Inflammation – The body’s natural response to injury or infection, often causing redness, heat, swelling, and pain.

Kynurenine pathway – A chemical process in the body involved in breaking down the amino acid tryptophan, often linked to immune responses.

Lung clearance – The way the lungs remove inhaled particles, usually by moving them out through mucus or by immune cells carrying them away.

Macrophage – A type of immune cell that “eats” harmful particles, bacteria, and dead cells.

Membrane remodeling – Changes to the structure or function of a cell’s outer layer (membrane).

Mutagenesis – The process of causing mutations or changes in DNA.

Oxidative stress – Damage caused by an imbalance between harmful molecules (free radicals) and the body’s ability to neutralize them.

Pro-inflammatory cytokine – A chemical released by immune cells that promotes inflammation.

Toxicity – The degree to which a substance can cause harm to living organisms by damaging cells, tissues, or organs, depending on the dose and exposure duration.

**Figure 5:**
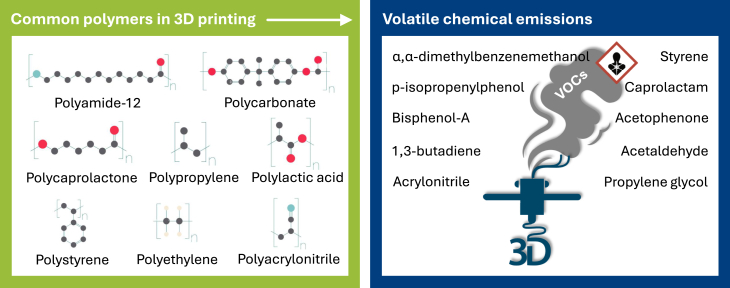
Examples of chemical emissions in polymer 3D printing Left panel: common polymer materials used in 3D printing; right panel: associated volatile chemical emissions. These emissions may act through various toxicological modes of action, including oxidative stress, endocrine disruption, and genotoxicity, highlighting potential risks linked to inhalation and dermal exposure during the printing process.

In addition to powder feedstocks and generated nanoparticles, 3D printing processes often utilize thermoplastic filaments as raw materials, which emit volatile organic compounds (VOCs), such as acrylonitrile-butadiene-styrene (ABS). ABS degrades at high temperatures into acrylonitrile, 1,3-butadiene, and styrene - all of which are toxic to humans.^103^^,^^104^ In polymer 3D printing during the polycarbonate (PC) filament print run, fused filament fabrication (FFF) 3D printers emit compounds tentatively identified as bisphenol A (BPA), *p*-isopropenylphenol and phenol, whereas styrene, 3-cyclohexen-1-ylbenzene, α,α-dimethylbenzenemethanol, and acetophenone were detected when ABS filament was used,^104^ as shown in Figure 5. BPA is a well-known endocrine disruptor with potential to induce carcinogenesis and mutagenesis in animal models.^105^^,^^106^ Some 3D printing-emitted aerosols contain harmful elements such as barium, chromium, and manganese as well as VOCs, like acetaldehyde, a potential occupational carcinogen, and 2-hydroxypropyl methacrylate, an immune sensitizer.^107^

Dermal exposure is generally mitigated by the skin’s barrier function, but nickel, chromium, and other metals found in industrial 3D printing can still pose risks. *In vitro* studies have shown that human keratinocytes accumulate nickel, cobalt, and especially hexavalent chromium to a high degree, leading to reduced cell viability.^108^ These metals are known to cause allergic contact dermatitis and may provoke cytotoxic and inflammatory responses even in the absence of visible skin damage.^109^^,^^110^ Additionally, data suggest that TiO_2_ particles can penetrate the skin, leading to systemic distribution and pathological changes in animal models.^111^ Ocular exposure to AM-generated particles, such as iron and TiO_2_ can cause severe damage, including inflammation.^112^

Due to their stability and poor solubility, many metal nanoparticles are not metabolized by the human body. For example, aluminum and other metal particles detected in brain tissues have been linked to neurological damage.^113^^,^^114^ Additionally, various exogenous particles have been detected in human cerebrospinal fluid, exhibiting prolonged retention in the brain compared to other organs raising the risks for long-term effects on the brain.^115^ Biomarkers like F2-isoprostane increase following exposure to metal oxide nanoparticles, correlating with higher risks of systemic diseases, such as coronary artery disease.^116^ Compounds released from PLA, such as lactide, propylene glycol, and caprolactam, are associated with ocular and respiratory toxicity.^117^^,^^118^^,^^119^ Since these and many other substances are formed or transformed by 3D printing, the information above, gathered from other types of settings, underscores the urgent need for comprehensive toxicological translation and evaluations and improved safety measures in 3D printing processes in order to mitigate potential health impacts.

Taken together, these findings reflect an urgent sustainability challenge, i.e., without a deeper understanding of the long-term health impacts of particle and chemical emissions in 3D printing, it becomes difficult to ensure the safe and responsible scaling of 3D printing. Sustainable industrial development must include frameworks that protect human health while supporting innovation, particularly as 3D printing becomes a cornerstone of future manufacturing.

## Home 3D printing: Comparable health risks on a larger scale

The increasing presence of 3D printers in home and public space (e.g., schools, libraries) environments raises significant concerns about indoor air quality (IAQ) due to the emission of PM and VOCs.^37^^,^^61^^,^^62^^,^^63^ The type, brand, and color of printing filaments have been shown to dramatically influence emission profiles, with emission rates varying by as much as 150 times depending on the filament brand.^120^ These emissions are primarily generated through the thermal degradation of materials, particularly in material extrusion printers, leading to the release of submicron and ultrafine particles that deposit in the lungs and may pose respiratory health risks.^16^^,^^121^^,^^122^

Among filament types, ABS is associated with significantly higher ultrafine particle emissions and respiratory symptoms compared to PLA filaments. A study of 26 healthy individuals exposed to ABS filament emissions reported symptoms such as weakness and bad breath, with slight increases in exhaled nitric oxide (FeNO) as a marker of eosinophilic inflammation.^123^ More recent research indicates that particle emissions can by far exceed earlier reported values. For example, García-González and López-Pola^124^ measured emissions from acrylonitrile styrene acrylate (ASA) filaments exceeding 1,700,000 particles/cm^3^, while Gu et al.^37^ reported emission rates from ASA of up to 1.7 × 10^11^ particles per minute.

Although chemical vapor emissions from 3D printing are generally lower in impact than particle emissions, styrene and ethylbenzene release from certain filaments remains a concern.^118^ Even though studies on particle and VOC emissions from desktop 3D printers exist,^37^^,^^61^^,^^62^^,^^63^ the current understanding of their health effects remains very limited, especially considering that polymers contain a range of chemical additives and residual monomers, which may be released or transformed during printing. This highlights an urgent need for further research into their potential health and environmental impacts.

## A framework for sustainable and safe 3D printing: Aligning innovation with human health

While this article highlights the need for policy action, it is important to note that comprehensive regulatory frameworks specific to 3D printing emissions remain largely undeveloped. In the EU, no dedicated guidelines currently exist for the safe use of 3D printing technologies in relation to VOC or particle emissions. Some national efforts have been made, for example recent guidance from the US National Institute for Occupational Safety and Health (NIOSH) addresses exposure risks in educational and public settings and proposes control strategies for airborne emissions (NIOSH, 2023),^52^ the Swedish Chemicals Agency (KEMI)^125^ provides safety recommendations for home and public use, and the Finnish Institute of Occupational Health (FIOH)^126^ has developed a practical control approach for managing chemical safety in occupational 3D printing environments. However, these initiatives are limited in scope and for the latter, not harmonized at the EU level. This lack of coordinated sector-specific regulation underscores the urgent need for harmonized, forward-looking policies to ensure safe and sustainable 3D printing practices.

In the rapidly evolving landscape of 3D printing, ensuring the safety of both workers and home users is essential. The diverse and dynamic nature of existing and emerging materials presents unique challenges that require an interdisciplinary framework for risk assessment. Developing such a framework for home settings is particularly difficult due to factors like printer type, materials used, printer placement, and ventilation. Therefore, this perspective focuses on industrial 3D printing and unintentional particle emissions. Other aspects such as VOC emissions, environmental consequences, and exposures in non-industrial settings are mentioned for contextual relevance, but they are not within the primary scope of the proposed framework. While these topics are of highest relevance and in need of dedicated investigation, here they are included to underscore the broader need for comprehensive safety strategies across all 3D printing contexts. To support general awareness and safer practices beyond industrial settings, practical recommendations for minimizing emissions in home and public settings are provided in Box 4.

Box 4 **Mitigating emissions in home and public space 3D printing: practical insights and recommendations**

While this article primarily focuses on industrial 3D printing, it is important to recognize that health risks also exist in home and public space environments, where tailored risk assessment frameworks are harder to apply. Several practical strategies can help mitigate exposure to emissions in these settings.

Measurement tools such as low-cost indoor air quality (IAQ) sensors, gas chromatography-mass spectrometry (GC-MS), and particle analyzers like WIBS (Wideband Integrated Bioaerosol Sensors) offer accessible means to monitor emissions. The WIBS model has proven particularly useful for distinguishing between polymer types and quantifying emissions from ABS filaments.^118^

Studies have emphasized the importance of emission control strategies. Viitanen et al.^127^ reported that both general ventilation and local exhaust ventilation with a canopy hood were insufficient for managing nanoparticle emissions from desktop 3D printers. However, a retrofitted enclosure reduced emissions by 96%, presenting an effective and practical solution. Similarly, Deng et al.^128^ found that ABS filaments emit significantly more nanoparticles than PLA, particularly during the heating phase. Notably, pre-heating the extruder and print bed reduced ABS emissions by up to 75%.

These findings underscore the need for a combination of emission monitoring tools, engineering controls, and user adherence to safety guidelines—especially when printing in poorly ventilated areas or producing objects intended for children or food use. In line with this, the Swedish Chemicals Agency (KEMI),^125^ in collaboration with the Swedish Work Environment Authority (Arbetsmiljöverket),^129^ has issued safety recommendations for minimizing exposure in home and public settings. These include placing printers in well-ventilated rooms, choosing safer filament types, and following best practices during operation (KEMI, 2024).^125^ The US National Institute for Occupational Safety and Health (NIOSH, 2023)^52^ also provides guidance for schools, libraries, and small businesses, recommending engineering controls such as enclosures and localized ventilation to minimize emissions during 3D printing.

**Figure 6:**
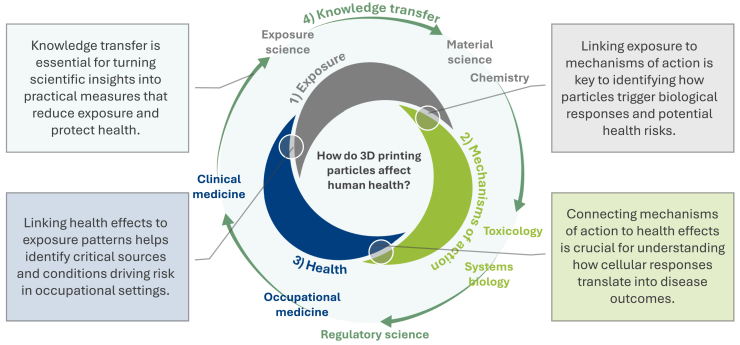
Toward a risk assessment framework Developing a robust framework for the 3D printing industry requires interdisciplinary collaboration and continuous knowledge flow and exchange across key disciplines.

**Figure 7:**
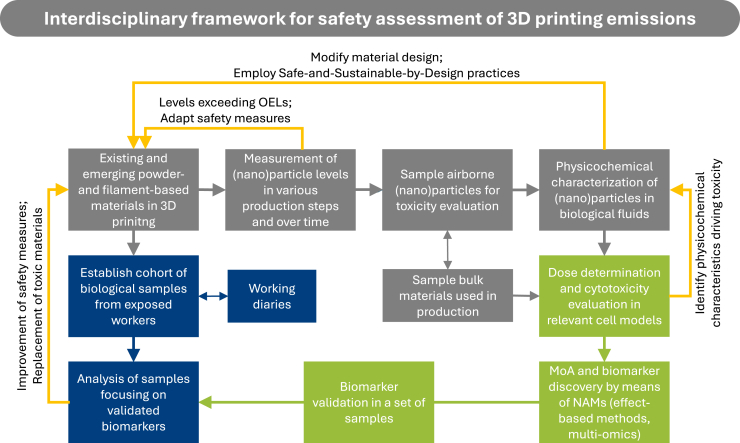
Interdisciplinary research framework for the risk assessment of particle emissions in industrial 3D printing The framework focuses on (1) exposure assessment by measuring particle emissions, and characterizing their physicochemical properties, and (2) hazard identification by assessing their effects on human cells and health, discovering health effect biomarkers, and establishing cohorts of exposed workers to provide a comprehensive understanding of 3D printing-associated health risks. OEL, Occupational Exposure Limits; MoA, Mode of Action; NAMs, New Approach Methodologies.

Building a robust risk assessment framework for the 3D printing industry requires synergy across multiple disciplines and continuous exchange of generated knowledge (Figure 6). We propose an interdisciplinary approach that integrates exposure assessment through particle emission measurements and characterization of physicochemical properties as well as hazard identification done by evaluating effects on human cells and health, identifying biomarkers of exposure and effect, and establishing cohorts of exposed workers for biomonitoring. This approach aims to advance a comprehensive understanding of 3D printing-related health risks and to support the development of safe-and-sustainable-by-design practices in industrial settings (Figure 7). It builds on the efforts and methodologies developed within the HÄMAT2-3 and NanoSafety1-2 projects (https://www.oru.se/english/research/projects/nt/nanosafety2/), funded by the Swedish Innovation Agency (Vinnova) and the Swedish Knowledge Foundation, in which we have been actively involved or governed. The proposed framework is intended as a general, adaptable model for assessing particle emissions and associated health effects in industrial 3D printing, which can be tailored to various research and occupational contexts. The case study included in the later section case study: a Swedish industrial 3D printing company, illustrates how the framework can be applied in practice.

### Step 1. Measurement of (nano)particle levels and exposure target levels

The first step in developing a comprehensive risk assessment framework involves the precise measurement of (nano)particle levels across all operational stages, including material preparation, active printing, post-processing (e.g., support removal, sanding, curing), equipment cleaning, and powder recycling, to capture both routine and incidental emissions over time. This data forms the basis for subsequent toxicity evaluations, health monitoring, and informs decision-making processes for implementing safety measures in industrial 3D printing. Given the continuous evolution of printing materials and technologies, along with the frequent emergence of new technologies that develop broad exposure limits, encompass various feedstock materials and 3D printing processes, it becomes increasingly complex. In addition, activities where feedstock powders are reused or reconditioned represent a potentially significant exposure source due to particle handling and re-aerosolization and should consequently be explicitly included in the exposure assessment strategy.

Currently, no established limit values exist for nanoparticles. However, the World Health Organization (WHO) has proposed global air quality guidelines for nanoparticles in ambient environments: 20,000 particles/cm^3^ (1-h mean) and 10,000 particles/cm^3^ (24-h mean) (WHO, 2021),^130^ offering useful reference points for both occupational and public exposure settings. For industrially generated nanoparticles, the Finnish Institute of Occupational Health (FIOH, 2021)^126^ has defined target exposure levels for an 8-h exposure period: 20,000 particles/cm^3^ (for particles with a density >6000 kg/m^3^) and 40,000 particles/cm^3^ (for particles with a density <6000 kg/m^3^). In addition, Swedish OEL for inhalable dust has a limit of 5 mg/m^3^ and respirable dust of 2.5 mg/m^3^ (Swedish Work Environment Authority, 2018).^129^

To support these monitoring efforts, a range of analytical tools, such as cascade impactors, condensation particle counters, and scanning mobility particle sizers, can be employed to quantify size-specific particle distributions and exposure levels at the workplace. To further promote methodological consistency, Tang^131^ has developed a standard emission test method for FFF 3D printing that uses a defined strand printing protocol, controlled environmental conditions, and technically specified instrumentation and printers, to enable reproducible assessment of particle emissions across filament products and printer models. Furthermore, to specifically assess metal exposure, filters or surface samples collected during air monitoring can be analyzed using techniques such as inductively coupled plasma mass spectrometry (ICP-MS) to quantify concentrations of metals like nickel, chromium, or titanium. In addition to particle measurement tools, environmental conditions such as facility size, ventilation type, airflow patterns, and printer placement should be documented and evaluated, as they significantly influence particle dispersion and worker exposure. These data are essential for linking exposure levels to health outcomes and identifying potential exceedances of OELs.

### Step 2. Toxicity assessment and physicochemical characterization in biological fluids

While establishing (nano)particle levels, the focus is also on toxicity evaluation. Samples of airborne particles are collected for utilization in in-depth *in vitro-ex vivo* toxicological analysis to determine their potential impact on human health. Concurrently, physicochemical characterization of these particles in biological fluids (e.g., artificial lung fluid, artificial sweat) are conducted to provide insights into their behavior at different exposure sites or upon intracellular uptake (e.g., behavior in artificial lysosomal fluid).^101^ Toxicity can be assessed using high-content screening, multi-omics methods, or gene reporter assays, while characterization may involve techniques such as dynamic light scattering, electron microscopy, and both surface and bulk compositional analysis, for example, using X-ray photoelectron spectroscopy to determine surface composition and inductively coupled plasma mass spectrometry (ICP-MS) to quantify metal ion release in simulated biological fluids. These methods provide critical insights into particle reactivity, which is a key factor influencing their toxicological profile in exposed workers. This interdisciplinary approach ensures a comprehensive understanding of potential hazards associated with specific materials used in industrial 3D printing and their respective emissions.

### Step 3. Identification of physicochemical characteristics driving toxicity

A central aspect of the framework is the identification of physicochemical characteristics that drive toxicity. Multivariate analysis and clustering approaches can be applied to correlate physicochemical parameters (e.g., size, shape, reactivity) with observed biological effects, helping to identify key drivers of toxicity. Understanding the underlying factors contributing to adverse health effects enables targeted interventions and modifications in the manufacturing process. This proactive approach ensures that safety measures evolve alongside technological advancements, maintaining a balance between innovation and risk mitigation.

### Step 4. Cohort establishment and working diaries

To achieve the framework’s applicability, a cohort of biological samples (e.g., blood, saliva, serum, urine, exhaled breath) from exposed 3D printing workers is established. This longitudinal approach monitors health outcomes over time. Working diaries maintained by employees help correlate potential exposure incidents with health-related events, facilitating a more nuanced understanding of risks associated with specific work tasks or production steps. Diary-based exposure mapping can be enhanced with wearable particle monitors to better understand personal exposure levels.

### Step 5. Health effect biomarker discovery and validation

The proposed framework advances beyond traditional toxicity assessments and clinical biomarkers by incorporating state-of-the-art techniques, such as effect-based methods and multi-omics for mechanistic understanding, with validation performed using matched control and exposed sample groups. This leads to the discovery of biomarkers in blood, urine, or exhaled breath that serve as indicators of exposure and potential health effects. Rigorous validation of these biomarkers using a set of exposed and non-exposed samples ensure reliability and precision in the assessment process. For instance, the phosphatidylcholine profile in exhaled breath from 3D printing workers handling liquid, powder, or filament-based plastic materials may indicate exposure impacts and serve as an early biomarker for respiratory effects.^132^

### Step 6. Analysis and improvement measures

The analysis phase focuses on evaluating collected cohort samples and validated biomarkers to assess the potential health effects and effectiveness of existing safety measures. If particle emissions exceed target levels (see step 1. Measurement of (nano)particle levels and exposure target levels), or if emissions from specific printing materials induce toxicity, either *in vitro* or in collected samples, urgent actions are to be taken to adopt safety measures. These actions may include reducing the amount of and/or exposure time for people working in processes involving high risks, improving personal protective equipment, replacing harmful materials, modifying material designs, or implementing Safe-and-Sustainable-by-Design practices. Risk mitigation can also be informed by root-cause analysis of emission sources and efficacy testing of personal protective equipment using controlled exposure chambers. To ensure these actions are effective, follow-up assessments should be conducted. This includes re-measuring particle levels and re-evaluating biological markers after implementing mitigation strategies such as enhanced ventilation, enclosure systems, or material substitutions. Comparing exposure and effect data before and after interventions allows for the estimation of mitigation efficacy and supports continuous improvement of workplace safety practices.

## Assessing the framework's fit to current challenges

The proposed risk assessment framework addresses several critical gaps identified in the challenge-framing section. While it represents significant advancement over existing fragmented approaches, there are areas where the framework’s limitations must be acknowledged and its adaptability further refined.

The framework introduces robust tools for real-time monitoring of particle emissions and in-depth physicochemical characterization, but its reliance on advanced techniques may limit its scalability in low-resource settings. For instance, the necessity of specialized equipment, like cascade air impactors, poses challenges for widespread adoption, particularly in small-scale industrial facilities or home environments where the need for safety measures is equally pressing, especially where emission volumes are not negligible.

The framework is designed with industrial 3D printing environments in mind, where exposure risks are typically more pronounced due to higher emission volumes. However, its application to home and educational settings requires further contextualization. For instance, emissions in home settings are influenced by variables such as ventilation quality, material type, and printer placement, which are not fully accounted for in the current iteration.

While the framework emphasizes toxicity testing in biological fluids and *in vitro* models, these approaches may not fully capture long-term, low-dose exposure effects, particularly relevant to home users and vulnerable populations, such as children and individuals with pre-existing health conditions. Longitudinal cohort studies and chronic exposure models could strengthen the framework’s applicability to these scenarios. The inclusion of multi-omics approaches for biomarker discovery and in identifying mode of action is an important strength in guiding *ex vivo* analyses and monitoring the impact caused by exposure on health over time. Yet the validation of identified biomarkers across diverse exposure scenarios remains a challenge. For example, respiratory biomarkers identified in workers handling powders vs. working on post-processing of printed products may be different. A tiered validation strategy that incorporates diverse exposure settings (e.g., specific work task, duration of exposure time, exposure levels) would enhance the framework’s robustness.

The framework’s alignment with existing regulatory guidelines, such as those proposed by the Finnish Institute of Occupational Health, provides a starting point for standardized assessments. However, its utility in influencing policy depends on its ability to integrate with and adapt to varying emerging guidelines, particularly in regions with limited occupational health infrastructures. Finally, while the framework represents a comprehensive approach, its complexity may pose barriers to adoption by small-scale industries and home users. Simplified, modular components, such as a basic emission profiling toolkit or a low-cost exposure assessment app, could help bridge the gap between the framework’s comprehensiveness and practical utility.

## Case study: A Swedish industrial 3D printing company

**Figure 8:**
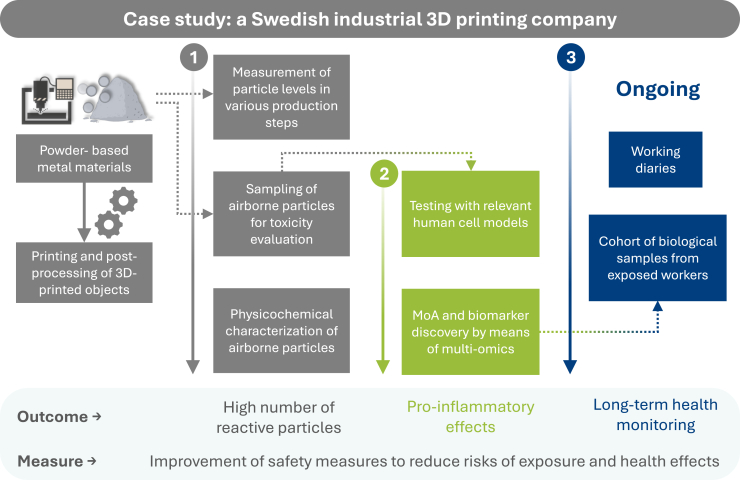
Implementation of the proposed risk assessment framework in a Swedish industrial 3D printing company This figure illustrates how the interdisciplinary framework is applied in an industrial setting, integrating particle exposure assessment, toxicological evaluation, and health monitoring. The approach enables the identification of exposure scenarios, relevant modes of action (MoA), and potential biomarkers, ultimately supporting safer and more sustainable 3D printing practices.

The ongoing projects in which the authors are involved provided an opportunity to apply the proposed risk assessment framework in a Swedish industrial 3D printing company, demonstrating its effectiveness in identifying and mitigating risks associated with particle exposure (Figure 8). It represents an early-stage implementation of the framework, which will serve as the foundation for several forthcoming publications. Following *Step 1* of the framework, particle measurements were conducted across various stages and processes, with the highest levels detected in the post-processing of 3D-printed parts (Dr. Lena Andersson, personal communication). In *Step 2*, the collected particles were characterized for their physicochemical properties (e.g., size distribution, shape, bulk chemical composition), revealing highly reactive nano- and microscale particles (Dr. Patrik Karlsson, personal communication). These findings informed *Step 3*, where the particles were tested on human cell models. The results demonstrated pronounced inflammatory responses, as well as metabolomic and transcriptomic changes indicative of inflammation (A.A., personal communication). In line with *Steps 4* and *5*, a worker cohort is being established with ongoing collection of blood, saliva, urine, and exhaled breath samples from workers to support long-term health monitoring and uncover potential biomarkers of exposure. Diaries are being collected from employees to record daily tasks, use, location, and time frame of safety equipment, potential exposure incidents, and health-related events. These records will help correlate exposure scenarios with health outcomes over time. Despite the use of protective clothing and masks, operating under industrial conditions can be challenging, especially when exposure involves invisible and airborne threats, such as NPs and VOCs. Results obtained in *Steps 1–5* and worker observations in the post-processing room revealed gaps in adherence to protection protocols, directly informing *Step 6* of the framework, which involves implementing and improving safety measures. This led to the introduction of stricter personal protective equipment guidelines with the aim of reducing risks for exposure and health effects.

This case study highlights the framework’s practical application in identifying exposure risks, characterizing hazards, and guiding targeted interventions to improve safety in 3D printing environments.

## Limitations and future directions

The proposed framework is specifically designed for assessing particle emissions in industrial 3D printing. It does not extend to home or public environments, where different exposure scenarios and safety conditions prevail. It also does not cover VOCs released during polymer 3D printing, which represent a significant and distinct class of emissions with potential respiratory and systemic health effects. Future work should integrate VOC exposure and toxicity into the framework to support a more comprehensive risk assessment strategy for industrial 3D printing.

In outlook, while 3D printing represents a promising advancement in sustainable manufacturing, it also poses potential health risks that must be carefully evaluated. Moreover, as 3D printing technology develops further, especially in home and school settings, understanding and preventing its impact on health is crucial in ensuring that its benefits are realized without compromising public well-being. Continued interdisciplinary research is essential to address these health and safety challenges, focusing on both exposure assessment and hazard identification. Based on the existing evidence concerning 3D printing, it is now time for immediate actions in order to mitigate the risks and ensure that the widespread adoption of 3D printing does not compromise health or environmental safety nor hamper industrial innovation and development. Therefore, to ensure that the expansion of 3D printing aligns with health and environmental protection goals, we offer the following recommendations: (1) for industry, implement safe-and-sustainable-by-design principles early in product and process development by integrating emission monitoring and substitution of hazardous materials; (2) for academia, prioritize interdisciplinary, long-term studies that capture both particle and VOC exposure effects across diverse 3D printing settings, particularly chronic low-dose impacts; (3) for regulatory agencies, establish harmonized, exposure-based guidelines and standard testing protocols for 3D printing emissions to ensure consistent risk assessment and occupational and public health protections; (4) for policymakers, support legislative and funding initiatives that drive research, innovation, and regulatory development in 3D printing safety to protect public health while promoting responsible technological advancement. With interdisciplinary approaches, we can mitigate emerging risks while promoting sustainable innovation.

## Acknowledgments

This work was supported by the Swedish Knowledge Foundation [grant nos. 20160019; 20220122, and 20230020], and Vinnova, the Swedish Agency for Innovation Systems, [grant no. 2021-03968]. We acknowledge scientific support from the Exploring Inflammation in Health and Disease (X-HiDE) Consortium, which is a strategic research profile at Örebro University funded by the Knowledge Foundation [grant no. 20200017].

## Author contributions

A.A., conceptualization, formal analysis, funding acquisition, investigation, project administration, visualization, supervision, writing – original draft, and writing – review and editing; D.S., formal analysis, writing – original draft, and writing – review and editing; M.E., funding acquisition, project administration, supervision, writing – review and editing. E.S., conceptualization, funding acquisition, project administration, supervision, writing – review and editing.

## Declaration of interests

The authors declare no competing interests.

## Declaration of generative AI and AI-assisted technologies in the writing process

During the preparation of this work, the author(s) used ChatGPT, a language model developed by OpenAI in order to improve readability of parts of the text. After using this tool/service, the author(s) reviewed and edited the content as needed and take(s) full responsibility for the content of the published article.

## References

[1] Lengua C.A.G., Farooqy K.M. (2017). Rapid prototyping in cardiac disease: 3D Printing the Heart.

[2] Mueller T., Elkaseer A., Charles A., Fauth J., Rabsch D., Scholz A., Marquardt C., Nau K., Scholz S.G. (2020). Eight weeks later—the unprecedented rise of 3D printing during the COVID-19 pandemic—a case study, lessons learned, and implications on the future of global decentralized manufacturing. Appl. Sci..

[3] Gebler M., Schoot Uiterkamp A.J.M., Visser C. (2014). A global sustainability perspective on 3D printing technologies. Energy Policy.

[4] Srivastava M., Rathee S. (2022). Additive manufacturing: Recent trends, applications and future outlooks. Prog. Addit. Manuf..

[5] Al Rashid A., Koç M. (2023). Additive manufacturing for sustainability and circular economy: needs, challenges, and opportunities for 3D printing of recycled polymeric waste. Mater. Today Sustain..

[6] Kassab A., Al Nabhani D., Mohanty P., Pannier C., Ayoub G.Y. (2023). Advancing plastic recycling: Challenges and opportunities in the integration of 3d printing and distributed recycling for a circular economy. Polymers.

[7] Olawumi M.A., Oladapo B.I., Ikumapayi O.M., Akinyoola J.O. (2023). Waste to wonder to explore possibilities with recycled materials in 3D printing. Sci. Total Environ..

[8] Hassan M., Mohanty A.K., Misra M. (2024). 3D printing in upcycling plastic and biomass waste to sustainable polymer blends and composites: A review. Mater. Des..

[9] Ghabezi P., Sam-Daliri O., Flanagan T., Walls M., Harrison N.M. (2024). Circular economy innovation: A deep investigation on 3D printing of industrial waste polypropylene and carbon fibre composites. Resour. Conserv. Recycl..

[10] STOA. 2021. Key enabling technologies for Europe's technological sovereignty. https://www.europarl.europa.eu/stoa/en/document/EPRS_STU(2021)697184.

[11] Phillips J.I., Green F.Y., Davies J.C.A., Murray J. (2010). Pulmonary and systemic toxicity following exposure to nickel nanoparticles. Am. J. Ind. Med..

[12] Ortega R., Bresson C., Darolles C., Gautier C., Roudeau S., Perrin L., Janin M., Floriani M., Aloin V., Carmona A., Malard V. (2014). Low-solubility particles and a Trojan-horse type mechanism of toxicity: the case of cobalt oxide on human lung cells. Part. Fibre Toxicol..

[13] Assenhöj M., Almstrand A.C., Kokelj S., Ljunggren S.A., Olin A.C., Karlsson H. (2023). Occupational exposure and health surveys at metal additive manufacturing facilities. Front. Public Health.

[14] Karlsson H.L., Vallabani N.V.S., Wang X., Assenhöj M., Ljunggren S., Karlsson H., Odnevall I. (2023). Health hazards of particles in additive manufacturing: a cross-disciplinary study on reactivity, toxicity and occupational exposure to two nickel-based alloys. Sci. Rep..

[15] Qi M., Wang X., Chen J., Liu Y., Liu Y., Jia J., Li L., Yue T., Gao L., Yan B. (2023). Transformation, absorption and toxicological mechanisms of silver nanoparticles in the gastrointestinal tract following oral exposure. ACS Nano.

[16] Portugal J., Bedia C., Amato F., Juárez-Facio A.T., Stamatiou R., Lazou A., Campiglio C.E., Elihn K., Piña B. (2024). Toxicity of airborne nanoparticles: Facts and challenges. Environ. Int..

[17] Donaldson K., Seaton A. (2012). A short history of the toxicology of inhaled particles. Part. Fibre Toxicol..

[18] Riediker M., Zink D., Kreyling W., Oberdörster G., Elder A., Graham U., Lynch I., Duschl A., Ichihara G., Ichihara S. (2019). Particle toxicology and health-where are we? Part. Fibre Toxicol..

[19] Zhang Y., Zhang Y., Que H., Lu C., Zhou S. (2025). Occupational nanoparticles: major sources, physicochemical properties, multi-organ toxic effects, and associated mechanisms. Toxicol. Mech. Methods.

[20] Luo X., Xie D., Hu J., Su J., Xue Z. (2022). Oxidative stress and inflammatory biomarkers for populations with occupational exposure to nanomaterials: A systematic review and meta-analysis. Antioxidants.

[21] Jia S., Setyawati M.I., Liu M., Xu T., Loo J., Yan M., Gong J., Chotirmall S.H., Demokritou P., Ng K.W., Fang M. (2022). Association of nanoparticle exposure with serum metabolic disorders of healthy adults in printing centers. J. Hazard. Mater..

[22] Zhao L., Zhu Y., Chen Z., Xu H., Zhou J., Tang S., Xu Z., Kong F., Li X., Zhang Y. (2018). Cardiopulmonary effects induced by occupational exposure to titanium dioxide nanoparticles. Nanotoxicol.

[23] Forest V., Pourchez J., Pélissier C., Audignon Durand S., Vergnon J.M., Fontana L. (2021). Relationship between occupational exposure to airborne nanoparticles, nanoparticle lung burden and lung diseases. Toxics.

[24] Mohammadian Y., Nasirzadeh N. (2021). Toxicity risks of occupational exposure in 3D printing and bioprinting industries: a systematic review. Toxicol. Ind. Health.

[25] Rodríguez-Hernández A.G., Chiodoni A., Bocchini S., Vazquez-Duhalt R. (2020). 3D printer waste, a new source of nanoplastic pollutants. Environ. Poll..

[26] Khosravani M.R., Reinicke T. (2020). On the environmental impacts of 3D printing technology. Appl. Mater. Today.

[27] Blok L.G., Longana M.L., Yu H., Woods B.K.S. (2018). An investigation into 3D printing of fibre reinforced thermoplastic composites. Addit. Manuf..

[28] Loke G., Yuan R., Rein M., Khudiyev T., Jain Y., Joannopoulos J., Fink Y. (2019). Structured multimaterial filaments for 3D printing of optoelectronics. Nat. Commun..

[29] Zhang J., Amini N., Morton D.A.V., Hapgood K.P. (2021). 3D printing with particles as feedstock materials. Adv. Powder Technol..

[30] Vaes D., Van Puyvelde P. (2021). Semi-crystalline feedstock for filament-based 3D printing of polymers. Prog. Polym. Sci..

[31] Arockiam A.J., Subramanian K., Padmanabhan R.G., Selvaraj R., Bagal D.K., Rajesh S. (2022). A review on PLA with different fillers used as a filament in 3D printing. Mater. Today: Proceedings.

[32] Min K., Li Y., Wang D., Chen B., Ma M., Hu L., Liu Q., Jiang G. (2021). 3D printing-induced fine particle and volatile organic compound emission: An emerging health risk. Environ. Sci. Technol. Lett..

[33] Tedla G., Jarabek A.M., Byrley P., Boyes W., Rogers K. (2022). Human exposure to metals in consumer-focused fused filament fabrication (FFF)/3D printing processes. Sci. Total Environ..

[34] Alijagic A., Engwall M., Särndahl E., Karlsson H., Hedbrant A., Andersson L., Karlsson P., Dalemo M., Scherbak N., Färnlund K. (2022). Particle safety assessment in additive manufacturing: From exposure risks to advanced toxicology testing. Front. Toxicol..

[35] Romanowski H., Bierkandt F.S., Luch A., Laux P. (2023). Summary and derived risk assessment of 3D printing emission studies. Atmos. Environ. X..

[36] Runström Eden G., Tinnerberg H., Rosell L., Möller R., Almstrand A.C., Bredberg A. (2022). Exploring methods for surveillance of occupational exposure from additive manufacturing in four different industrial facilities. Ann. Work Expo. Health.

[37] Gu J., Wensing M., Uhde E., Salthammer T. (2019). Characterization of particulate and gaseous pollutants emitted during operation of a desktop 3D printer. Environ. Int..

[38] Das S., Bourell D.L., Babu S.S. (2016). Metallic materials for 3D printing. MRS Bull..

[39] Schraufnagel D.E. (2020). The health effects of ultrafine particles. Exp. Mol. Med..

[40] Moreno-Ríos A.L., Tejeda-Benítez L.P., Bustillo-Lecompte C.F. (2022). Sources, characteristics, toxicity, and control of ultrafine particles: An overview. Geosci. Front..

[41] Andersson L., Bryngelsson I.L., Hedbrant A., Persson A., Johansson A., Ericsson A., Lindell I., Stockfelt L., Särndahl E., Westberg H. (2019). Respiratory health and inflammatory markers-Exposure to respirable dust and quartz and chemical binders in Swedish iron foundries. PLoS One.

[42] Andersson L., Hedbrant A., Persson A., Bryngelsson I.L., Sjögren B., Stockfelt L., Särndahl E., Westberg H. (2021). Inflammatory and coagulatory markers and exposure to different size fractions of particle mass, number and surface area air concentrations in the Swedish hard metal industry, in particular to cobalt. Biomarkers.

[43] Hedbrant A., Andersson L., Bryngelsson I.L., Eklund D., Westberg H., Särndahl E., Persson A. (2020). Quartz dust exposure affects NLRP3 inflammasome activation and plasma levels of IL-18 and IL-1Ra in iron foundry workers. Mediators Inflamm..

[44] Hedbrant A., Eklund D., Andersson L., Bryngelsson I.L., Persson A., Westberg H., Särndahl E. (2022). Effects on white blood cell counts and the NLRP3 inflammasome due to dust and cobalt exposure in the hard metal industry. Biomarkers.

[45] Cheriyan D., Khamraev K., Choi J.H. (2021). Varying health risks of respirable and fine particles from construction works. Sustain. Cities Soc..

[46] Steinle S., Reis S., Sabel C.E., Semple S., Twigg M.M., Braban C.F., Leeson S.R., Heal M.R., Harrison D., Lin C., Wu H. (2015). Personal exposure monitoring of PM2. 5 in indoor and outdoor microenvironments. Sci. Total Environ..

[47] Braakhuis H.M., Park M.V.D.Z., Gosens I., De Jong W.H., Cassee F.R. (2014). Physicochemical characteristics of nanomaterials that affect pulmonary inflammation. Part. Fibre Toxicol..

[48] Xuan L., Ju Z., Skonieczna M., Zhou P.K., Huang R. (2023). Nanoparticles-induced potential toxicity on human health: applications, toxicity mechanisms, and evaluation models. MedComm.

[49] Donaldson K., Poland C.A. (2012). Inhaled nanoparticles and lung cancer–what we can learn from conventional particle toxicology. Swiss Med. Wkly..

[50] Habas K., Demir E., Guo C., Brinkworth M.H., Anderson D. (2021). Toxicity mechanisms of nanoparticles in the male reproductive system. Drug Metab. Rev..

[51] 3D Printing Industry (2024). https://3dprintingindustry.com/news/half-million-3d-printers-sold-2017-track-100m-sold-2030-131642/#:%7E:text=Following%203%20years%20of%20strong%20growth%20%282015-2017%29%20it,printers%20would%20reach%20over%2050M%20units%20in%202030.

[52] Hodson L., Dunn K.L., Dunn K.H., Glassford E., Hammond D., Roth G., Cincinnati O.H., NIOSH (2023). U.S. Department of Health and Human Services, Centers for Disease Control and Prevention, National Institute for Occupational Safety and Health.

[53] Chen R., Yin H., Cole I.S., Shen S., Zhou X., Wang Y., Tang S. (2020). Exposure, assessment and health hazards of particulate matter in metal additive manufacturing: A review. Chemosphere.

[54] Zontek T.L., Ogle B.R., Jankovic J.T., Hollenbeck S.M. (2017). An exposure assessment of desktop 3D printing. J. Chem. Health Saf..

[55] Colnot E., Cardoit L., Cabirol M.J., Roudier L., Delville M.H., Fayoux A., Thoby-Brisson M., Juvin L., Morin D. (2022). Chronic maternal exposure to titanium dioxide nanoparticles alters breathing in newborn offspring. Part. Fibre Toxicol..

[56] Kumah E.A., Fopa R.D., Harati S., Boadu P., Zohoori F.V., Pak T. (2023). Human and environmental impacts of nanoparticles: a scoping review of the current literature. BMC Public Health.

[57] Yan Z., Liu Z., Yang B., Zhu X., Song E., Song Y. (2024). Long-term pulmonary iron oxide nanoparticles exposure disrupts hepatic iron-lipid homeostasis and increases plaque vulnerability in ApoE−/− mice. Environ. Pollut..

[58] Yi J., Duling M.G., Bowers L.N., Knepp A.K., LeBouf R.F., Nurkiewicz T.R., Ranpara A., Luxton T., Martin S.B., Burns D.A. (2019). Particle and organic vapor emissions from children’s 3-D pen and 3-D printer toys. Inhal. Toxicol..

[59] Sripada K., Wierzbicka A., Abass K., Grimalt J.O., Erbe A., Röllin H.B., Weihe P., Díaz G.J., Singh R.R., Visnes T. (2022). A children’s health perspective on nano-and microplastics. Environ. Health Perspect..

[60] House R., Rajaram N., Tarlo S.M. (2017). Case report of asthma associated with 3D printing. Occup. Med..

[61] Stephens B., Azimi P., El Orch Z., Ramos T. (2013). Ultrafine particle emissions from desktop 3D printers. Atmos. Environ. X..

[62] Azimi P., Zhao D., Pouzet C., Crain N.E., Stephens B. (2016). Emissions of ultrafine particles and volatile organic compounds from commercially available desktop three-dimensional printers with multiple filaments. Environ. Sci. Technol..

[63] Mendes L., Kangas A., Kukko K., Mølgaard B., Säämänen A., Kanerva T., Flores Ituarte I., Huhtiniemi M., Stockmann-Juvala H., Partanen J. (2017). Characterization of emissions from a desktop 3D printer. J. Ind. Ecol..

[64] Kühnel D., Steska T., Schlich K., Wolf C., Wohlleben W., Hund-Rinke K. (2023). Polymers of low concern? Assessment of microplastic particles used in 3D printing regarding their toxicity on *Raphidocelis subcapitata* and *Daphnia magna*. Micropl. Nanopl..

[65] Oladapo B.I., Bowoto O.K., Adebiyi V.A., Ikumapayi O.M. (2023). Net zero on 3D printing filament recycling: A sustainable analysis. Sci. Total Environ..

[66] Alijagic A., Suljević D., Fočak M., Sulejmanović J., Šehović E., Särndahl E., Engwall M. (2024). The triple exposure nexus of microplastic particles, plastic-associated chemicals, and environmental pollutants from a human health perspective. Environ. Int..

[67] Villarrubia-Gómez P., Carney Almroth B., Eriksen M., Ryberg M., Cornell S.E. (2024). Plastics pollution exacerbates the impacts of all planetary boundaries. One Earth.

[68] Lee J.Y., An J., Chua C.K. (2017). Fundamentals and applications of 3D printing for novel materials. Appl. Mater. Today.

[69] Shahrubudin N., Lee T.C., Ramlan R. (2019). An overview on 3D printing technology: Technological, materials, and applications. Procedia Manuf..

[70] Jandyal A., Chaturvedi I., Wazir I., Raina A., Ul Haq M.I. (2022). 3D printing–A review of processes, materials and applications in industry 4.0. Sustain. Oper. Comp..

[71] Khan S.A., Jassim M., Ilcan H., Sahin O., Bayer İ.R., Sahmaran M., Koc M. (2023). 3D printing of circular materials: Comparative environmental analysis of materials and construction techniques. Case Stud. Constr. Mater..

[72] Elhadad A.A., Rosa-Sainz A., Canete R., Peralta E., Begines B., Balbuena M., Alcudia A., Torres Y. (2023). Applications and multidisciplinary perspective on 3D printing techniques: Recent developments and future trends. Mat. Sci. Eng. R: Rep..

[73] Fonseca N., Thummalapalli S.V., Jambhulkar S., Ravichandran D., Zhu Y., Patil D., Thippanna V., Ramanathan A., Xu W., Guo S. (2023). 3D printing-enabled design and manufacturing strategies for batteries: A review. Small.

[74] Garg M., Rani R., Meena V.K., Singh S. (2023). Significance of 3D printing for a sustainable environment. Mater. Today Sustain..

[75] Liu Z., Zhang M., Bhandari B., Wang Y. (2017). 3D printing: Printing precision and application in food sector. Trends Food Sci. Technol..

[76] Baigarina A., Shehab E., Ali M.H. (2023). Construction 3D printing: A critical review and future research directions. Prog. Addit. Manuf..

[77] McMahon M. (2024). https://www.metal-am.com/articles/metal-powders-in-additive-manufacturing-an-exploration-of-sustainable-production-usage-and-recycling/?utm_source=chatgpt.com.

[78] Nazir A., Gokcekaya O., Billah K.M.M., Ertugrul O., Jiang J., Sun J., Hussain S. (2023). Multi-material additive manufacturing: A systematic review of design, properties, applications, challenges, and 3D printing of materials and cellular metamaterials. Mat. Des..

[79] Tan C., Li R., Su J., Du D., Du Y., Attard B., Chew Y., Zhang H., Lavernia E.J., Fautrelle Y. (2023). Review on field assisted metal additive manufacturing. Int. J. Mach. Tools Manuf..

[80] Ma T., Zhang Y., Ruan K., Guo H., He M., Shi X., Guo Y., Kong J., Gu J. (2024). Advances in 3D printing for polymer composites: A review. InfoMat.

[81] Oliveira J.P., LaLonde A.D., Ma J. (2020). Processing parameters in laser powder bed fusion metal additive manufacturing. Mater. Des..

[82] Chowdhury S., Yadaiah N., Prakash C., Ramakrishna S., Dixit S., Gupta L.R., Buddhi D. (2022). Laser powder bed fusion: a state-of-the-art review of the technology, materials, properties & defects, and numerical modelling. J. Mater. Res. Technol..

[83] Petrovic V., Vicente Haro Gonzalez J., Jordá Ferrando O., Delgado Gordillo J., Ramón Blasco Puchades J., Portolés Griñan L. (2011). Additive layered manufacturing: sectors of industrial application shown through case studies. Int. J. Prod. Res..

[84] King W.E., Anderson A.T., Ferencz R.M., Hodge N.E., Kamath C., Khairallah S.A., Rubenchik A.M. (2015). Laser powder bed fusion additive manufacturing of metals; physics, computational, and materials challenges. Appl. Phys. Rev..

[85] Hopkinson N., Hague R., Dickens P. (2006).

[86] Chen Z., Li Z., Li J., Liu C., Lao C., Fu Y., Liu C., Li Y., Wang P., He Y. (2019). 3D printing of ceramics: A review. J. Eur. Ceram. Soc..

[87] Berman B. (2012). 3-D printing: The new industrial revolution. Bus. Horiz..

[88] Gomes J.F., Miranda R.M., Oliveira J.P., Esteves H.M., Albuquerque P.C. (2019). Evaluation of the amount of nanoparticles emitted in LASER additive manufacture/welding. Inhal. Toxicol..

[89] Bau S., Rousset D., Payet R., Keller F.X. (2020). Characterizing particle emissions from a direct energy deposition additive manufacturing process and associated occupational exposure to airborne particles. J. Occup. Environ. Hyg..

[90] Väisänen A.J.K., Hyttinen M., Ylönen S., Alonen L. (2019). Occupational exposure to gaseous and particulate contaminants originating from additive manufacturing of liquid, powdered, and filament plastic materials and related post-processes. J. Occup. Environ. Hyg..

[91] Rejeski D., Zhao F., Huang Y. (2018). Research needs and recommendations on environmental implications of additive manufacturing. Addit. Manuf..

[92] Semmler M., Seitz J., Erbe F., Mayer P., Heyder J., Oberdörster G., Kreyling W.G. (2004). Long-term clearance kinetics of inhaled ultrafine insoluble iridium particles from the rat lung, including transient translocation into secondary organs. Inhal. Toxicol..

[93] Chen R., Chen C. (2012). The Nanobiotechnology Handbook.

[94] Renwick L.C., Brown D., Clouter A., Donaldson K. (2004). Increased inflammation and altered macrophage chemotactic responses caused by two ultrafine particle types. Occup. Environ. Med..

[95] Zisook R.E., Simmons B.D., Vater M., Perez A., Donovan E.P., Paustenbach D.J., Cyrs W.D. (2020). Emissions associated with operations of four different additive manufacturing or 3D printing technologies. J. Occup. Environ. Hyg..

[96] Rokni M.R., Nutt S.R., Widener C.A., Champagne V.K., Hrabe R.H. (2017). Review of relationship between particle deformation, coating microstructure, and properties in high-pressure cold spray. J. Ther. Spray Tech..

[97] Miller A., Drake P.L., Hintz P., Habjan M. (2010). Characterizing exposures to airborne metals and nanoparticle emissions in a refinery. Ann. Occup. Hyg..

[98] Wang Y., Chen L., Chen R., Tian G., Li D., Chen C., Ge X., Ge G. (2017). Effect of relative humidity on the deposition and coagulation of aerosolized SiO_2_ nanoparticles. Atmos. Res..

[99] Alijagic A., Scherbak N., Kotlyar O., Karlsson P., Wang X., Odnevall I., Benada O., Amiryousefi A., Andersson L., Persson A. (2023). A novel nanosafety approach using cell painting, metabolomics, and lipidomics captures the cellular and molecular phenotypes induced by the unintentionally formed metal-based (nano) particles. Cells.

[100] Vallabani N.V.S., Alijagic A., Persson A., Odnevall I., Särndahl E., Karlsson H.L. (2022). Toxicity evaluation of particles formed during 3D-printing: Cytotoxic, genotoxic, and inflammatory response in lung and macrophage models. Toxicol.

[101] Alijagic A., Wang X., Vallabani N.V.S., Melin P., Särndahl E., Karlsson H.L., Odnevall I. (2024). Characteristics and health risks of the inhalable fraction of metal additive manufacturing powders. Nano Select.

[102] Alijagic A., Kotlyar O., Larsson M., Salihovic S., Hedbrant A., Eriksson U., Karlsson P., Persson A., Scherbak N., Färnlund K. (2024). Immunotoxic, genotoxic, and endocrine disrupting impacts of polyamide microplastic particles and chemicals. Environ. Int..

[103] Secondo L.E., Adawi H.I., Cuddehe J., Hopson K., Schumacher A., Mendoza L., Cartin C., Lewinski N.A. (2020). Comparative analysis of ventilation efficiency on ultrafine particle removal in university MakerSpaces. Atmos. Environ..

[104] Farcas M.T., Stefaniak A.B., Knepp A.K., Bowers L., Mandler W.K., Kashon M., Jackson S.R., Stueckle T.A., Sisler J.D., Friend S.A. (2019). Acrylonitrile butadiene styrene (ABS) and polycarbonate (PC) filaments three-dimensional (3-D) printer emissions-induced cell toxicity. Toxicol. Lett..

[105] Ahmad I., Kaur M., Tyagi D., Singh T.B., Kaur G., Afzal S.M., Jauhar M. (2024). Exploring novel insights into the molecular mechanisms underlying Bisphenol A-induced toxicity: A persistent threat to human health. Environ. Toxicol. Pharmacol..

[106] Ma Y., Liu H., Wu J., Yuan L., Wang Y., Du X., Wang R., Marwa P.W., Petlulu P., Chen X., Zhang H. (2019). The adverse health effects of bisphenol A and related toxicity mechanisms. Environ. Res..

[107] Bowers L.N., Stefaniak A.B., Knepp A.K., LeBouf R.F., Martin S.B., Ranpara A.C., Burns D.A., Virji M.A. (2022). Potential for exposure to particles and gases throughout vat photopolymerization additive manufacturing processes. Buildings.

[108] Ermolli M., Menné C., Pozzi G., Serra M.Á., Clerici L.A. (2001). Nickel, cobalt and chromium-induced cytotoxicity and intracellular accumulation in human hacat keratinocytes. Toxicol.

[109] Ahlström M.G., Thyssen J.P., Wennervaldt M., Menné T., Johansen J.D. (2019). Nickel allergy and allergic contact dermatitis: A clinical review of immunology, epidemiology, exposure, and treatment. Contact Dermat..

[110] Yoshihisa Y., Shimizu T. (2012). Metal allergy and systemic contact dermatitis: an overview. Dermatol. Res. Pract..

[111] Wu J., Liu W., Xue C., Zhou S., Lan F., Bi L., Xu H., Yang X., Zeng F.D. (2009). Toxicity and penetration of TiO_2_ nanoparticles in hairless mice and porcine skin after subchronic dermal exposure. Toxicol. Lett..

[112] Eom Y., Song J.S., Lee H.K., Kang B., Kim H.C., Lee H.K., Kim H.M. (2016). The effect of ambient titanium dioxide microparticle exposure to the ocular surface on the expression of inflammatory cytokines in the eye and cervical lymph nodes. Investig. Ophthalmol. Vis. Sci..

[113] Krewski D., Yokel R.A., Nieboer E., Borchelt D., Cohen J., Harry J., Kacew S., Lindsay J., Mahfouz A.M., Rondeau V. (2007). Human health risk assessment for aluminium, aluminium oxide, and aluminium hydroxide. J. Toxicol. Environ. Health, Part B.

[114] Maher B.A., Ahmed I.A.M., Karloukovski V., MacLaren D.A., Foulds P.G., Allsop D., Mann D.M.A., Torres-Jardón R., Calderon-Garciduenas L. (2016). Magnetite pollution nanoparticles in the human brain. Proc. Natl. Acad. Sci. USA.

[115] Qi Y., Wei S., Xin T., Huang C., Pu Y., Ma J., Zhang C., Liu Y., Lynch I., Liu S. (2022). Passage of exogeneous fine particles from the lung into the brain in humans and animals. Proc. Nat. Ac. Sci. USA.

[116] Milne G.L., Yin H., Hardy K.D., Davies S.S., Roberts L.J. (2011). Isoprostane generation and function. Chem. Rev..

[117] Davis A.Y., Zhang Q., Wong J.P.S., Weber R.J., Black M.S. (2019). Characterization of volatile organic compound emissions from consumer level material extrusion 3D printers. Build. Environ..

[118] Finnegan M., Thach C.L., Khaki S., Markey E., O'Connor D.J., Smeaton A.F., Morrin A. (2023). Characterization of volatile and particulate emissions from desktop 3D printers. Sensors.

[119] Potter P.M., Al-Abed S.R., Hasan F., Lomnicki S.M. (2021). Influence of polymer additives on gas-phase emissions from 3D printer filaments. Chemosphere.

[120] Khaki S., Duffy E., Smeaton A.F., Morrin A. (2021). Monitoring of particulate matter emissions from 3D printing activity in the home setting. Sensors.

[121] Farmer D.K., Vance M.E., Abbatt J.P.D., Abeleira A., Alves M.R., Arata C., Boedicker E., Bourne S., Cardoso-Saldaña F., Corsi R. (2019). Overview of HOMEChem: House observations of microbial and environmental chemistry. Environ. Sci. Process. Impacts.

[122] Zhou Y., Kong X., Chen A., Cao S. (2015). Investigation of ultrafine particle emissions of desktop 3D printers in the clean room. Proc. Eng..

[123] Gümperlein I., Fischer E., Dietrich-Gümperlein G., Karrasch S., Nowak D., Jörres R.A., Schierl R. (2018). Acute health effects of desktop 3D printing (fused deposition modeling) using acrylonitrile butadiene styrene and polylactic acid materials: An experimental exposure study in human volunteers. Indoor Air.

[124] Garcia-Gonzalez H., Lopez-Pola M.T. (2024). Unlocking the nanoparticle emission potential: a study of varied filaments in 3D printing. Environ. Sci. Poll. Res..

[125] KEMI (2024). https://www.kemi.se/rad-till-privatpersoner/kemikalier-i-hemmet-och-pa-fritiden/3d-skrivare.

[126] FIOH (2021). https://www.ttl.fi/teemat/tyoturvallisuus/altistuminen-tyoympariston-haittatekijoille/kemiallisten-tekijoiden-hallinta-tyopaikalla/tyoympariston-riskienhallinnan-malliratkaisut.

[127] Viitanen A.K., Kallonen K., Kukko K., Kanerva T., Saukko E., Hussein T., Hämeri K., Säämänen A. (2021). Technical control of nanoparticle emissions from desktop 3D printing. Indoor Air.

[128] Deng Y., Cao S.J., Chen A., Guo Y. (2016). The impact of manufacturing parameters on submicron particle emissions from a desktop 3D printer in the perspective of emission reduction. Build. Environ..

[129] Arbetsmiljöverket författningssamling (AFS 2018:1). Hygieniska gränsvärden. (2018). Arbetsmiljöverket.

[130] World Health Organization (2021). https://apps.who.int/iris/handle/10665/345329.

[131] Tang C.L. (2025). https://depositonce.tu-berlin.de/handle/11303/24302.

[132] Almstrand A.C., Bredberg A., Runström Eden G., Karlsson H., Assenhöj M., Koca H., Olin A.C., Tinnerberg H. (2023). An explorative study on respiratory health among operators working in polymer additive manufacturing. Front. Public Health.

